# How can we meet the needs of patients, their families and their communities? A qualitative study including clinicians, consumer representatives, patients, and community members

**DOI:** 10.1186/s12913-023-09814-9

**Published:** 2023-07-28

**Authors:** Natasha Roberts, Helene Jacmon, Brighid Scanlon, Chrissy Battersby, Peter Buttrum, Christine James

**Affiliations:** 1 University of Queensland Centre for Clinical Research, Butterfield St, Herston, QLD 4029 Australia; 2 STARS Education and Research Alliance, Surgical Treatment and Rehabilitation Service (STARS), The University of Queensland and Metro North Health, Herston, Australia; 3 Royal Brisbane and Women’s Hospital, Herston, Australia; 4 School of Public Health, Queensland University Technology, Kelvin Grove, QLD Australia; 5 The University of Queensland, St Lucia, QLD Australia

**Keywords:** Health services, Diversity, Unmet needs, Screening, Transition care, Assessment, Equity

## Abstract

**Background:**

The *Diversity Working Group* was formed in response to Australian Quality and Safety Health Care Standards that require organisations plan service delivery that incorporates information about the diversity of consumers, and those at higher risk of harm.

**Methods:**

A qualitative gap analysis was conducted by a team from varied professional backgrounds including a clinician researcher, a nurse researcher with expertise in culturally and linguistically diverse care and a consumer representative with expertise in advocacy and carer representation. Qualitative questions were co-designed, using a person-centred care lens. Community organisation members, and clinicians and patients from both ambulatory and inpatient areas were approached. Responses were coded independently and synthesised using a Framework Methodology.

**Results:**

In total 3 community organisation members, 40 clinicians and 30 patients consented to participate in the qualitative study over a period of three weeks. There were three key themes across responses, ‘*What are diverse needs?*’; ‘*Assigning people to a group does not address a need*’; ‘*Unplanned care makes people feel vulnerable’*. Those patients who are isolated, for any number of reasons, were identified as at greater risk of harm.

**Conclusion:**

Taking a person-centred approach can potentially better understand the needs of patients and communities so that this information can be incorporated into health service delivery. Resources are needed to support patients and their families at times of transition care, particularly when care is unplanned.

**Supplementary Information:**

The online version contains supplementary material available at 10.1186/s12913-023-09814-9.

## Key implications

- A person centred approach can potentially meet needs across patient groups, especially those at risk of harm

- Transitioning between services was a time when patients feel most vulnerable

- Quality care that meets the needs of patients, families and communities requires multi-layered approach supported by the health service, the local clinical area and with local clinical teams.

**Supplementary Information:**

The online version contains supplementary material available at 10.1186/s12913-023-09814-9.

## Background

Delivering healthcare across all patient populations is a goal of many high-income countries, including Australia [1]. However, healthcare services have historically provided universalistic, “one-size-fits-all” approach to service provision and patient engagement [2]. It is well recognised that this approach leads to entrenched unmet healthcare needs, and potential harm, for specific patients whose needs may place them at greater risk of harm [3]. This often stems from the lack of information incorporated in health service delivery about diverse consumers accessing services, particularly those that are marginalised when facing economic or cultural barriers [4]. In Australia, the Australian National Safety and Quality Health Service Standards (v2) [5], require organisations to identify the “diverse needs of their consumers and communities…and those at higher risk of harm” to plan and deliver care accordingly [5, 6]. In response, the *Diversity Working Group* was established to evaluate and improve service provision for patients within the largest public hospital health service in Australia.

A rapid review of the literature confirmed that being a part of a specific population in itself does not put a person and/or their community at risk of harm, however, the literature acknowledged that health services often do not reflect the needs of many populations [7–10]. State and federal government resources highlighted that if these needs are ignored, health inequities may result [10]. Internationally and nationally, there was a call for targeted and culturally responsive healthcare to address disparities and promote health equity [7–21]. At a national level, there were five publications identifying health determinants, alongside enablers and barriers to service delivery [14–19]. Two publications reported programs that included care coordination and supported navigation of health services for those groups identified as at risk of harm [18, 19]. However, there was a paucity of research providing evaluations or evidence-based guidance for health services to meet the diverse needs of patients. To redress this, the *Diversity Working Group* initiated a qualitative gap analysis to answer the question: *How can diverse needs of patients and communities be met in our hospital health service?*

## Methods

As a first step, experts across the organisation were engaged to identify patient priority groups, especially those at greater risk of harm, to align with the Australian National Safety and Quality Health Service Standards. These experts were recommended by the quality and safety team and executive team. The diverse groups identified as potentially at risk of harm were Aboriginal and Torres Strait Islander peoples, Culturally and Linguistically Diverse (CALD) people, those living with a disability (physical or cognitive), patients from rural/ remote areas, people experiencing homelessness or social disadvantage, those with a mental health issue, and people who identify as Lesbian, Gay, Bisexual, Transgender, Queer, intersex and other sexuality, gender, and bodily diverse people and communities (LGBTQI +).

### Aims of the project

This qualitative project was designed to understand any gaps in service provision. We proposed that this exploration would assist the *Diversity Working Group* plan future directions and service delivery. A *Diversity Officer* role was funded, and the full-time equivalent was divided into three positions, including two nurses with clinical and qualitative research expertise and a consumer advisor with expertise in advocacy and health ethics (NR, BS, HJ). Those in the role personally identified with a diverse group.

### Theoretical approach

By consensus, the team determined that diverse needs could be assessed using a person-centred care approach, defined by the Australian Commission on Safety and Quality in Healthcare as being “…respectful of, and responsive to, the preferences, needs and values of patients and consumers” [5]. This term was largely used for feasibility, as there is no standardised definition or framework for the term *‘diverse*’ identified from the literature. Assessing the needs of the identified populations through the person-centred care approach allowed an evidence-based framework to be applied whilst supporting the research question.

The three *Diversity Officers* co-designed sets of qualitative open-ended questions, to ask of clinicians, patients, and community groups. Qualitative questions for clinicians aimed to explore enablers and barriers for meeting needs, to ensure “person-centered care” of patients and their communities. Qualitative questions for patients also aimed to better explore what the term ‘*diverse needs’* meant to patients, what ‘diverse’ groups they identified with, and the perceived enablers and barriers for having their needs met. The sets of qualitative questions were disseminated to members of the *Diversity Working Group* for feedback and improvement.

### Recruitment of participants

After permissions were sought from local managers, patients and carers were approached in both outpatient and inpatient areas to participate. The project was explained, the voluntary and confidential nature of participation was reinforced, and the independent role of *Diversity Officers* to clinical areas. Clinical staff were invited to participate at unit meetings, and the qualitative questions were also emailed to clinicians to capture those that worked outside standard business hours. Other patient facing services, such as social work, allied health, discharge facilitation were approached by email. Community groups identified by patients, clinicians, and the broader working group, were contacted by telephone. Verbal consents were obtained, and no identifying information was collected about participants. Recruitment continued until the funded time frame expired.

### Data collection

*Diversity Officers* asked participants the codesigned questions, and documented responses on a printed data collection form which had each question listed. Responses were written down with the participant present. After each interview, additional thoughts were documented on the data collection form as a memo. Interviews were one on one and took 10 min time on average.

### Data analysis

Over three weeks, the *Diversity Officers* collated all responses to the qualitative questions. A Framework Method was used to organise and synthesise the findings [22]. Each *Diversity Officer* inductively assigned codes and constructs independently. These were put into matrices when the *Diversity Officers* then came together and synthesised further. Memos were used to better understand any divergent data and commonalities across themes. Findings were discussed in two rounds, at which time consensus was achieved. After a 12-month time-period, a final revision of the data and themes was repeated in preparation of this manuscript. COREQ guidelines were used for reporting [23].

### Ethics

A submission was finalised with the Royal Brisbane and Women’s Hospital Human Research Ethics Committee (LNR/2019/QRBW/54984) prior to study commencement.

## Results

In total three community organisation group members from primary care networks, 40 clinicians and 30 patients consented to participate in the qualitative study over a period of three weeks. There were three key themes bringing together the responses. These were titled, ‘*What are diverse needs?*’; ‘*Assigning people to a group does not identify a need*’; ‘*Unplanned care makes people feel vulnerable’*.

### What are diverse needs?

There were varying opinions as to how clinical teams identify the diverse needs of patients and families. Some clinicians reported that they identified ‘*diverse’* needs by directly asking patients, and/or families and carers on admission. Others reported that any needs were identified by reviewing clinical notes and by asking carers of patients. Whilst some clinicians said that simply asking patients would be the best way to identify their needs, others reported that they felt uncomfortable asking patients to ‘pigeonhole’ themselves. *“…you are asking people to tell you how they are different; it is a hard question…”* (clinician) and *“…some people carry trauma about this sort of stuff…”* (clinician).

Additionally, patients reported their own discomfort declaring any variation from what is “normal”: *“…I don’t know about the groups, I don’t know what the answer means for me*” (patient). Another patient expressed concern about the potential implications from their response, *“People don’t want to be ‘different’”* (patient). Existing organisational structures to communicate any specific needs were identified at various timepoints on the patient journey, including on admission (Risk assessment, Booking form, emergency department presentations), during care (Structured Interdisciplinary Bedside Rounding (SIBR), Multidisciplinary teams (MDTs), and at discharge (discharge facilitators, referrals, follow-up appointments, general practitioner letters).

Interestingly, when asked directly, no patient participants identified with any of the identified priority groups, despite clinicians identifying them as such. Instead, patients in interviews reflected on their own health experiences, not a group they may identify with. For example, one patient who had an interpreter responded:

*“I have bad veins. I don’t like it when they are rude, I get scared they will be rude. I have no veins, I cannot help it, they keep poking thinking one will appear. I tell them. It hurts, and they get angry at me because I have no veins. That only happens every now and then, mostly everyone is very friendly, but it makes me feel shy” *(patient)

There were responses across all participant groups on the challenges of identifying ‘*diverse* needs’, and that they could be either overt or invisible. Both clinicians and patients raised concerns about relying on appearances at the risk of more pressing needs that may put patients at greater risk of harm: *“…you can’t see diversity”* (community organisation member).

Some participants expressed that the umbrella term *‘diversity’* was tokenistic and lacked commitment to addressing patient needs in day-to-day care. They reported past experiences contributing to other pieces of work that did not result in any observed changes within the health service: *“We see this sometimes, diversity, what is that? it’s tokenistic”* (community organisation member). Other clinical team members said that they were pleased that there was more attention on the diverse patient groups within the hospital community and looked forward to more resources for support.

### Assigning people to a group does not identify a need

Assigning patients to a priority group, such as homelessness, as a mechanism to identify any needs was reported to be problematic by clinicians. Some participants said that this had the potential to generate unwarranted assumptions and bias based on the group a patient may be assigned, rather than addressing specific health needs. In each clinical area, clinicians appeared to be aware of resources available for patients, but also acknowledged that there were also gaps in necessary resources. Each ward or department appeared to present unique patient groups, unique resources, and unique service needs and expressed frustration at the barriers to accessing more resources they perceived were used routinely in other institutions.

Patients described how they actively worked hard to build relationships with clinicians caring for them, that this was most important. For some patients, limitations with health service delivery resulted in a breakdown in trust. For example, patients said that waiting for long periods in the waiting room made them feel uncared for, that clinicians would avoid them: “*…ignoring you because you are inconvenient* (patient).

Conversely, there were many patients who reported that they were very happy with their care, that they have no concerns, that there was nothing to be improved, and that their needs are always met. As a result, they said they trusted clinicians would know what they needed. It appeared that patients who said that they did not trust their team, who vigilantly monitored what was happening, struggled to engage in their healthcare. Clinicians also reported that patient engagement was essential, and sometimes needed extra effort. They gave examples of when they would actively seek out those who they thought were struggling to engage with the hospital service: “*…you got to persist, don’t give up on anyone”* (nurse). Another clinician spoke about the importance of adjusting service delivery so they could better meet individual patient needs:

*“There was this lady, she was really anxious…we changed her appointments so that they worked for her, did extra follow-ups. My manager is very supportive. Being flexible was what helped her anxiety”.*

(clinician)

Patient reports reinforced that this was important, that they felt cared for when they saw clinicians fight for them: *“…when the staff fight for you”* (patient). Also, it was strongly reported that some patients wanted to have a voice in healthcare decisions: *“…taking on what I say, taking it on board.”* (patient). Patients explained that they felt *“cared for”* if clinicians took time to listen to them: *“…I saw an Obstetrician who took the time to really listen and explain; caring like a mother and I really appreciate that”* (patient). Others expressed frustration at not feeling like a person:

*“We are not customers or consumers… We are people with families that love us. We are loved by people. Not someone coming in to buy something. It is like they don’t care about who we are”.*

(patient)

Consistently, clinicians identified that patients who were at greater risk of harm were isolated, for whatever reason, such as social, cultural, or spatial reasons. For example, those who are from CALD backgrounds, those from rural and remote locations, young mothers with a history of addiction without family supports. Isolation resulted in inequities that came from difficulties accessing information and to physically access services in the hospital:

*“I don’t have a car, so normally I just would not go, its hard with all the kids on the bus. We make it work. I have had to learn to do things for myself, by myself, you learn to do it on your own. You just don’t know to ask for help, you actually don’t know how”.*

(patient)

Those with mental health issues were identified as those with a pressing need. Patients that experienced mental health barriers could be from any priority group and often had difficulties engaging with health services generally. There were also patients identified as falling between services, with unmet needs. Examples included those patients that do not have a Medicare card, or those awaiting assessment or approval for services such as National Disability Insurance Scheme or My Aged Care support. These groups had been identified as having specific health needs but attending to these was put on hold while they were waiting for a place in a supported service.

### Unplanned care makes people feel vulnerable

Transitioning between teams or services, whether it be planned or unplanned appeared to be a time when *‘diverse needs’* were amplified. Patients recounted experiences of when their health status changed, or there was a change of plan. It was a time when they said they felt vulnerable and isolated:

*“…changing the plan without telling you. Little things are like pulling pieces out of the puzzle. I know what it is like, I get it, but sometimes you wonder what else you have missed, is everything ok?”*

(patient)

Patients said that they did not want their lack of understanding and knowledge to be a barrier in decision making: *“…and be patient with me, sometimes I don’t understand everything the first time”* (patient). To be included was considered essential in decision-making: “*…when I am a member of the team”* (patient). Patients spoke of feeling frustrated about communication: “*…sometimes they communicate to themselves but not to the patients. I see them talking to each other in the corridor…”* (patient).

Patients pointed out that decisions had implications for not just them but also for their supports:

*“All I care about is my family, my partner and my child. When the plan changes, it’s on them. It means where I will go, what will I have to do, what will they have to do.”*

(patient)

Stories about healthcare experiences from patients sought to justify perceived needs, and why patients monitored their care closely to mitigate unexpected future events. For example, waiting for investigation results, was described as a time when patients could not advocate for themselves: *“It is hard to make decisions without all the information.”* (patient).

## Discussion

The findings reported here aim to address a call to identify the ‘*diverse* *needs*’ of patients and their communities in our hospital health service. We used existing frameworks for person-centred care, as there was not a standardised definition for ‘*diverse* needs’ available. We attempted to provide a more nuanced understanding across many populations, rather than homogenising them. By using such an approach, we inadvertently brought attention to the tensions when health service delivery focusses on “diversity” and “at risk” groups, when patients and families want to be seen as a person in their day-to-day interactions with clinical teams,

This work identified key areas that a large hospital health service can target to better address the needs of patients and community, including patient assessment, health care planning and transition care. These touchpoints are particularly important for those who are isolated, as they were found to be at higher risk of harm. There were varying ways clinicians reported that they identify the needs of the patients in their care, and that patients rarely fall into the well-defined categories. Patients in this study reported that they were best equipped when they were in a trusting relationship with their health care providers, which they characterised by being heard and feeling cared about. Those who were isolated struggled to engage with services, for a number of reasons. Across participant groups, it was acknowledged that needs may be invisible, and what may appear to be a priority to clinical teams may not reflect patients’ priorities. A key and novel finding of this work was that unplanned care and points of transition are when *‘diverse needs’* have the greatest impact.

Studies investigating the characteristics of successful organisations have identified that the acknowledgment of needs, and facilitation of a flexible approach to service delivery is both practical and effective. This was consistent with our findings where clinicians felt that they were able to provide optimal care when a flexible approach was supported [24, 25]. The literature has identified that patients’ needs are met when strategies are responsive to their preferences and values, so decisions support what is important to patients. Staff training is identified as an important enabler [24–33].

Evidence also supports our findings identifying that when planned healthcare changes, patients and their families felt most vulnerable [25, 33]. For transition care to be effectively provided, the literature identifies that services should be coordinated using robust care pathways and appropriate referrals, as it is the point in the patient’s journey of care when a cohesive multi-disciplinary team is most needed [34]. When unplanned changes happen for patients with complex and unique needs, transitions between providers or healthcare settings often result in fragmentation, low value care and poor patient outcomes [25, 35]. Such outcomes include functional decline, adverse events, adverse medication events, unplanned hospital admissions and patient dissatisfaction. Established strategies to respond include anticipating needs, providing actionable information and the provision of uninterrupted care, with unmet needs and under-utilisation of services a key barrier [34, 36]. Mitchell and colleagues reported that transition care could be optimised by ensuring patients had a sense of feeling cared for, being accountable and optimising collaboration between teams [34]. Velligan and colleagues report that key facilitators come with structured organisational support to ensure training and an adaptable approach [28]. Our study results support a layered strategy for tailoring care to individual patient needs, accommodated at multiple levels of a health service.

The literature tells us that both patient outcomes and health service outcomes are improved when we provide quality care at a patient level [37]. A needs assessment at a population level would likely identify the capacity of a health service to ensure adequate processes are in place [37]. Ensuring reliable cohesion in services for individual patients is a gap in evidence [38–41]. Drawing on our findings, organisational strategies could include implementation of shared decision-making structures, including patient reported outcome measures and an organisational philosophy that aligns with person-centred care [25, 27]. Shared decision making has been demonstrated to improve patient and carer engagement to positively impact patient outcomes [28].

How we move from talking about person centred care to creating structures that are operationalised day to day appears to be challenging [40, 42–46]. Those organisations that are reported to have the best quality patient experience data also have the highest quality and safety outcomes [38, 47].

Directions for future work should aim to better understand how to predict and understand needs, not just relying on heuristic clinician assessment alone. As a priority, more work is needed to better identify and understand the experiences of those who struggle to engage with health services. A national definition that encompasses ‘*diverse needs*’ or indeed, a more nuanced conceptualisation of those who may be structurally vulnerable within health services and institutions is lacking. It is imperative that a clear definition is comprehensively used throughout Australia so that equitable care provision can be readily assessed and compared, without promoting generalisations. Future research directions should aim to better understand how to use data to better model those who are isolated. Such an endeavour may ensure appropriate resources are readily available for clinical teams to better anticipate and respond directly to the needs of patients, their families, and their communities.

### Strengths and limitations

A strength of this study was its co-designed, qualitative approach. The use of the frameworks for person-centred care can be seen as both a strength and a limitation in this work. Whilst using this standardised definition and framework worked well and appeared to be a solution to the apparent challenges arising from the term ‘*diverse’,* there is a chance that another framework may have produced different results and implications. Responses were collected from both clinical inpatient and ambulatory spaces, including a wide breadth of participants who are engaged with day-to-day patient care day. These participants brought to light some of the strategies in place day to day within the hospital health service. However, it is likely that there are people with unmet needs who struggle to engage with health services did not participate in this study, as they may not be accessing or connecting with the health service, therefore, these findings are limited to those who are at least somewhat engaged with the health service. Organisational executive and health administrators were also not included in this study, but this is an area where much of the current literature is focussed.

The lack of demographic information about participants is an important limitation of this work.

## Conclusion

Taking a person-centred approach can potentially ensure that the ‘*diverse* needs’ of patients and communities will be better identified and responded to. Resources are needed to support patients and their families at times of transition care, particularly when care is unplanned.

## Supplementary Information

**Additional file 1.** Diversity in Health Care: A Gap Analysis.

## Data Availability

The datasets used and/or analysed during the current study are available from the corresponding author on reasonable request.

## Abbreviation

LGBTIQ +: Lesbian, Gay, Bisexual, Trans, intersex, Queer and other sexuality, gender, and bodily diverse people and communities
