# Supplementary material for: How can we meet the needs of patients, their families and their communities? A qualitative study including clinicians, consumer representatives, patients, and community members
Source: BMC Health Serv Res. 2023 Jul 28;23:809. doi: 10.1186/s12913-023-09814-9 (PMC10385916; doi:10.1186/s12913-023-09814-9)
Supplement: Supplementary file 1 — Additional file 1. Diversity in Health Care: A Gap Analysis. [file 12913_2023_9814_MOESM1_ESM.docx]

**Diversity in Health Care: A Gap Analysis**

**Questions**

1. Questions asked of staff and community stakeholders
2. What are the barriers to person-centred care of diverse patient populations?
3. What are the enablers to person-centred care of diverse patient populations?
4. Do you think there are any health service gaps in caring for diverse patient populations?
5. Do you think there are any patients from diverse groups at risk because their unique needs are not being met?
6. Questions asked of patients
7. What is important to you when you come to hospital?
8. Do you know what person-centred care means? (this term may need to be explained). What would this look like for you?
9. How can we make that happen?
10. What would stop that from happening?
11. Do you feel you have any unique needs?
12. You only need to answer yes or no once at the end of the complete list, but do you identify as any of the following:

- Aboriginal or Torres Strait Islander, someone from a remote or rural area, someone who is culturally or linguistically diverse, someone who is homeless or suffering financial hardship, someone living with a mental illness, someone who is older, someone who is younger, someone who is LGBTIQ+, someone who has a disability?

1. If so, do you think this makes a difference to the care you receive?
